# Nurse Educators’ Self-Reported Level of Teaching Competence and Its Correlation with Sociodemographic, Professional, Training and Research Variables: A Cross-Sectional Multicentre Study

**DOI:** 10.3390/nursrep16020041

**Published:** 2026-01-27

**Authors:** Isabel Martínez-Sánchez, Marta Romero-García, Sergio Alonso-Fernández, Maria-Antonia Martínez-Momblan, Judith Lleberia, Montserrat Puig-Llobet

**Affiliations:** 1 Fundación Hospital de l’Esperit Sant, 08923 Santa Coloma de Gramenet, Barcelona, Spain; 2 Fundamental and Clinical Nursing Department, Faculty of Nursing, University of Barcelona, 08007 Barcelona, Barcelona, Spain; salonso@ub.edu (S.A.-F.); mmartinezmo46@ub.edu (M.-A.M.-M.); 3 GRIN Research Group, IDIBELL, Bellvitge Biomedical Research Institute, 08908 L’Hospitalet de Llobregat, Barcelona, Spain; 4 Head of the Department of Gynaecology, Fundació Hospital de l’Esperit Sant, 08923 Santa Coloma de Gramenet, Barcelona, Spain; 5 Department of Public Health, Mental Health and Maternal-Child Nursing, Faculty of Nursing, Health Science Campus Bellvitge, University of Barcelona, 08907 L’Hospitalet de Llobregat, Barcelona, Spain; monpuigllob@ub.edu; 6 Research Group in Mental Health, Psychosocial and Complex Nursing Care (NURSEARCH), Faculty of Medicine and Health Sciences, University of Barcelona, 08007 Barcelona, Barcelona, Spain

**Keywords:** teaching competence, tutor, nurse teacher, nursing faculty, clinical placement, professional development, S-CONE

## Abstract

**Background:** Nurses’ teaching skills in the clinical setting are crucial to ensuring that students receive high-quality training. Despite the growing importance of competency frameworks, there is little research on the relationship between nurses’ teaching competence and sociodemographic, professional, training, and research variables. **Methods**: This was a cross-sectional, descriptive, and correlational study conducted at nine hospitals linked to the clinical placement subjects of the Bachelor of Nursing of the University of Barcelona. The study population comprised all nurses directly involved in clinical teaching. Participants’ level of self-reported teaching competence was evaluated using the Spanish version of the Capabilities of Nurse Educators (S-CONE) questionnaire, and the sociodemographic, professional, and academic variables were collected in an ad hoc questionnaire. Descriptive statistics, non-parametric tests, and linear and logistic regression models were used to analyse the associations between the S-CONE total score and the variables collected. **Results**: The mean age of the participants (n = 596) was 41.9 years (standard deviation: 8.82), and 85.6% of them were women (n = 510). The overall mean S-CONE score was 3.81 (SD: 0.73). Higher scores were observed in those with advanced academic degrees, formal teacher training, and participation in academic activities. Professionals with mixed roles (clinical mentor and academic tutor) self-reported significantly higher competence levels. Multivariate analyses identified participation in conferences, tutoring of undergraduate theses, and involvement in research or development projects as the main predictors of higher teaching competence as measured by the S-CONE questionnaire. The lowest-scoring factor was research and evidence, which points to a potential area for improvement. No significant associations were found with age, sex, or years of clinical experience. **Conclusions**: Participants had a high self-reported level of teaching competence and rated themselves as competent overall, especially in professional practice and curriculum design. However, we identified areas for improvement related to pedagogical innovation and the use of evidence. The findings reinforce the importance of professional development and academic involvement to strengthen teacher competence.

## 1. Introduction

Developing teaching skills in nursing is essential for preparing professionals who can respond effectively to the changing demands of health systems. This issue has gained international relevance since the Bologna Declaration, which promotes comparable educational standards across the European Union and addresses the challenges of modern healthcare. Within this global agenda, the World Health Organization (WHO) has defined a basic framework to help health educators to facilitate learning, effective communication, leadership, curriculum design, and the integration of evidence into practice [1]. These competencies represent the specific skills that enable educators to support student development in increasingly complex and transformative clinical settings. In line with these principles, a broader, more aspirational vision of teaching competence has been proposed, which incorporates leadership, innovation, and evidence-based practice within a holistic professional framework [2].

The term ‘capabilities’, conceptualised by McAllister and Flynn, reflects this broader vision of professional competence [2]. Rather than focusing solely on technical skills or observable behaviours, this perspective emphasises the educators’ agency, aspirations, and potential to innovate within their institutional context. Drawing on the Sen and Nussbaum capabilities approach [3], this framework considers what educators can do and can become in their professional roles, emphasising the importance of context, autonomy, and professional development.

In nursing training, clinical settings are considered the cornerstone of learning. Therefore, it is essential that educators can build bridges between theory and practice to ensure safe, high-quality care. Clinical educators must master technical skills and possess pedagogical and leadership abilities in order to effectively guide students and encourage critical thinking. However, transitioning from clinical practice to teaching roles can be challenging, particularly for professionals lacking formal pedagogical training [4]. Various studies have emphasised the importance of structured competency development, mentoring and a culture of evidence-based practice in order to boost self-confidence and professional commitment [5]. Such programmes align with international recommendations, supporting the integration of innovative practices, transformative leadership and enhanced teaching capabilities [6,7]. Effective communication and interdisciplinary collaboration are also critical to fostering inclusive learning environments and ensuring the successful integration of students into clinical teams [8]. In Spain, undergraduate nursing education includes a substantial number of mandatory clinical placements in care settings within the public and publicly funded health system. Clinical nurses play a key role in tutoring and supervising students in these settings. In Catalonia, an autonomous region in north-east Spain with a decentralised healthcare system and a high concentration of university hospitals in the Barcelona metropolitan area, nursing students’ clinical placements mainly take place in these hospitals, with close coordination between the university and healthcare services. Despite the importance of this role, few studies have systematically analysed the teaching competence of nurses involved in clinical education in Spain. To the best of our knowledge, no multicentre studies have used international teaching competence frameworks as a reference [1,9]. This lack of empirical evidence hinders the development of clinical faculty programmes and the establishment of institutional policies aimed at enhancing teaching in clinical practice. Despite advances in competency frameworks, little research has explored how teaching competence is perceived in relation to educators’ demographic, professional, training and academic profiles, particularly in southern European contexts where practical training models and the role of the clinical teacher differ considerably from those in English-speaking countries [1,9]. While researchers have studied the relationship between variables such as educational role and professional experience and perceived teaching capabilities [10,11,12,13], the associations between teaching competence and various professional variables have not yet been analysed using a multivariate approach. However, the associations between teaching competence and various professional variables have not yet been analysed using a multivariate approach. Building on the capabilities approach and the teaching competence framework underlying the Capabilities of Nurse Educators (CONE) questionnaire, we argue that teaching competence depends not only on individual attributes, but also on the opportunities and resources available in the professional environment [2]. We therefore conceive sociodemographic variables (such as age and years of experience), professional variables (clinical and teaching role, type of institution), training variables (specific education in teaching and clinical practice), and research-related variables (participation in projects, publications, conference communications) are, therefore, conceived as factors that can expand or constrain nurse educators’ capabilities to plan, implement, and evaluate high-quality learning experiences in clinical placements. This model informs the selection of the study variables and the interpretation of the multivariate analyses presented herein. To address the knowledge gap, we employed the Capabilities of Nurse Educators (CONE) questionnaire. This was developed McAllister and Flynn [2] and was adapted and validated in Spanish within the scope of this project (S-CONE). This tool enables a comprehensive evaluation of teaching competence in clinical settings. The objective of this study was to describe the level of competence of nurses with teaching responsibilities in clinical practice, as well as to analyse their relationship with sociodemographic, professional, training, and research variables, to identify the factors associated with a high level of self-reported teaching competence.

## 2. Materials and Methods

### 2.1. Design

This was a cross-sectional, prospective, and descriptive correlational study.

### 2.2. Context and Participants

The setting was the Faculty of Nursing of the University of Barcelona and nine university hospitals affiliated with the clinical placement subjects of the Bachelor of Nursing degree: Hospital Universitari de Bellvitge, Hospital Universitari Mútua de Terrassa, Fundació Hospital de l’Esperit Sant, Hospital Universitari Germans Trias i Pujol, Consorci Sanitari Integral, Hospital Universitari del Sagrat Cor, Hospital Universitari de Viladecans, Badalona Serveis Assistencials, and Fundació Sanitària Mollet. In total, ten institutions participated. The collection took place between May 2021 and September 2022.

### 2.3. Sample and Sampling

The study population was composed of two profiles of professionals with teaching responsibilities in the clinical placement subjects: (1) clinical mentors of the affiliated hospitals, who supervised students in the healthcare centres and (2) nurse educators from the Faculty of Nursing of the University of Barcelona, including nurse teachers, subject coordinators, and academic tutors. Academic tutors are faculty members who monitor and evaluate student learning during clinical placements and act as a liaison between the university and the healthcare centres, in collaboration with clinical mentors. The study included clinical mentors, nurse teachers, academic tutors, and nurses with a mixed role (clinical mentor and academic tutor).

Convenience sampling was used. For sample size estimation, the total population of nursing professionals involved in undergraduate clinical training at the participating institutions was estimated 1885 in January 2021. In the absence of previously published distribution parameters for the S-CONE total score, a standard deviation of 15 points was assigned as a conservative estimate of variability reported in clinical teaching competence measures used in nurse preceptors (e.g., SD = 16 in Wu et al.) [14] and following standard methodological recommendations for estimating a population mean in finite populations [15]. With a 95% confidence level (Z = 1.96) and a desired accuracy of ±3 points, the minimum required sample was 396 participants. This estimate was adjusted for an anticipated non-response rate of 20%. A total of 611 questionnaires were returned. Of these, 15 were excluded due to insufficient teaching experience or incomplete data, resulting in a final sample of 596 participants.

### 2.4. Instruments

#### 2.4.1. Ad Hoc Questionnaire

An ad hoc self-administered questionnaire was used to collected information on participants’ sociodemographic, professional, training, and research characteristics. Sociodemographic variables included age (years) and sex (male/female). Professional variables included years of experience as a nurse and as a clinical educator, employer, clinical unit (unidentified, hospitalization, emergency, intensive care, surgery, or other) and teaching role (clinical mentor, academic tutor, nurse teacher, or mixed role). The training variables included academic level (Diploma of Nursing, Bachelor’s degree, Master’s degree or Doctorate), receipt of teaching awards, and specific training related to teaching and clinical education. Research-related variables included academic and scientific activities conducted in the previous five years, such as supervision of undergraduate thesis, publications, conference presentations, authorship of book chapters or books, and participation in research, teaching, or the quality improvement projects.

#### 2.4.2. S-CONE Instrument

Teaching competence was assessed using the S-CONE, the validated Spanish version of the CONE questionnaire developed by McAllister and Flynn [2]. The S-CONE consists of 69 items grouped into five factors: nursing practice; curriculum design and implementation; communication, collaboration, and partnership; management, leadership and promotion; and research and evidence. Items are rated on a five-point Likert scale (1 = not representative, 5 = very representative), with higher scores indicting higher self-reported teaching competence. The total score ranges from 69 to 345. In this study, the S-CONE showed excellent internal consistency (Cronbach’s α = 0.96). Previous validation of the Spanish version demonstrated adequate temporal stability (test–retest ICC > 0.80), good construct validity, and a five-factor structure consistent with the theoretical model. Detailed information on the exploratory factor analysis, including factor loadings and eigenvalues, in provided in the Supplementary Material. Teaching competence scores were calculated as mean item scores for each dimension and for the total scale. For descriptive purposes, mean item score ≥ 3.0, corresponding to the theoretical midpoint of the Likert scale, was used to indicate a satisfactory level of self-reported teaching competence. The Spanish version (S-CONE) comprises 69 items retained after exploratory factor analysis. For traceability, item-level results are reported using the original CONE item identifiers (e.g., item 93 refers to CONE item 93 retained in S-CONE).

### 2.5. Data Collection Procedures

Data collection took place after the completion of students’ clinical placements. The two questionnaires were administered either in paper format or online via Google Forms (Google LLC, Mountain View, CA, USA). Both formats included an information sheet about the study and an informed consent form. For the paper-bases administration, academic tutors explained the study procedures to participants, distributed the questionnaires to clinical mentors in the participating healthcare centers, and subsequently collected the completed forms, which were returned to the research team through the subject coordinators. An online option was provided due to COVID-19 related restrictions. In this case, participants accessed the questionnaire through a link distributed by the teaching departments of the participating hospitals and, in the case of the University of Barcelona, by the subject coordinators. Paper questionnaires were returned in batches and contained no identifying information. Each participant generated a personal alphanumeric code used solely for management purposes (e.g., to avoid duplicate entries) and not for identification, ensuring anonymity and confidentiality during data processing and the analysis.

### 2.6. Ethical Considerations

The research complied with the fundamental bioethical principles established in the World Medical Association’s Declaration of Helsinki (2013). The study was approved by the Clinical Research Ethics Committee of Fundació Unió Catalana d’Hospitals (CEIm CI 18/30) on 8 June 2018 and by the Bioethics Committee of the University of Barcelona on 11 March 2018. In accordance with Spanish legislation on personal data protection (Organic Law 3/2018 of 5 December on the Protection of Personal Data and the Guarantee of Digital Rights), confidentiality and anonymity were ensured. Only members of the research team had access to the data. All participants received written information about the study and provided written informed consent prior to participation. Participation was voluntary, and participants were informed of their right to withdraw at any time without any consequences. They were also informed that the results would be used exclusively for the purposes stated in the research objectives. Data were collected in a pseudonymised form using an alphanumeric code generated from non-directly identifying variables. No direct personal identifiers were collected. The code was used exclusively to manage or avoid duplicate entries and not to identify participants.

### 2.7. Statistical Analysis

Descriptive statistics were used to summarise participants’ sociodemographic, professional, training, and research characteristics, as well as total scores and S-CONE score and its five dimensions. Data distribution was assessed using the Kolmogorov–Smirnov test. As most variables were not normally distributed, non-parametric tests were applied: the Mann–Whitney U test for comparisons between two groups and the Kruskal–Wallis test for three or more groups. Spearman’s rank correlation was used to examine associations between ordinal variables.

Multiple linear regression models were constructed to explore the associations between the S-CONE total score and the theoretically grouped predictors, organised into blocks: sociodemographic characteristics, professional experience, training, and research. Additionally, a binary logistic regression model was developed using a dichotomized outcome variable based on the 50th percentile of the S-CONE score.

The selection and structuring of the predictors were guided by previous studies identifying personal and professional variables associated with teaching competence in clinical settings, such as age, teaching role, professional experience, training and teacher self-efficacy [12,16,17,18]. In addition, the CONE questionnaire, the theoretical basis of the S-CONE, proposes a holistic approach to competence that integrates leadership, pedagogical innovation, and academic commitment [2]. Model fit for the logistic regression was assessed using the Hosmer–Lemeshow test, and model performance was evaluated using the area under the receiver operating characteristic curve (AUC). All analyses were performed using IBM SPSS Statistics (v29.0, IBM Corp., Armonk, NY, USA), and statistical significance was set at *p* < 0.05.

## 3. Results

A total of 596 nurse educators participated in the study, of whom 85.6% (n = 510) were female. The average age was 41.9 years, and most participants had more than 10 years of clinical experience. Regarding their teaching role, 81.4% (n = 485) were clinical mentors, and 10.2% (n = 61) performed a mixed role (clinical mentor and academic tutor). Nearly half held a master’s degree (47.8%), and the majority (73.8%) had not received formal teacher training. Further details of the participants’ characteristics are presented in Table 1.

### 3.1. Self-Reported Teaching Competence Levels

The mean item score of the S-CONE questionnaire was 3.81 (SD: 0.73). Detailed item-level results are presented in Supplementary Table S1, where items are classified into two categories: competent and not competent.

Overall, high levels of perceived competence were observed across all five S-CONE factors, although slightly lower scores were found for the research and evidence dimension. The highest self-reported competence was observed in items related to reflective practice, positive interpersonal attitudes, and recognition of the importance so research for clinical practice.

In contrast, comparatively lower levels of competence were observed in items related to stimulating student interest, identifying areas for improvement in teaching, applying theoretical frameworks, and maintaining interprofessional or academic networks. These patterns suggest that while participants perceived themselves as highly competent in relational, elective, and professional responsibility domains, they reported greater difficulty in areas related to pedagogical innovation, academic integration, and research-based teaching practices.

### 3.2. Relationship Between Teaching Competence and Sociodemographic, Professional, Training, and Research Characteristics

The bivariate analysis revealed statistically significant differences in the total S-CONE score across several professional, training and academic variables (see Table 2). Higher self-reported competence was observed among clinical nurses with advanced academic qualifications, those who had completed training in general education, and those who had participated in pedagogical training activities. Participants with a mixed role (clinical mentor and academic tutor) or solely an academic tutoring role reported higher levels of competence than those with exclusively clinical or teaching roles. Furthermore, involvement in academic and research-related activities, such as participating in projects, supervising undergraduate theses, presentations at conferences, and publishing articles, was associated with higher levels of self-reported teaching competence.

No significant differences were found according to sex, age, years of experience as a nurse or educator, or book publications. Differences were also observed between institutions and clinical units, with higher levels of self-reported competence reported by participants employed at tertiary hospitals settings.

Multiple linear regression analysis revealed that variables related to active academic and teaching engagement accounted for the largest proportion of variance in the S-CONE total score (adjusted R^2^ = 0.170), followed by educational role (adjusted R^2^ = 0.094). After adjustment, supervision of undergraduate theses, participation in scientific conferences and involvement in development or research projects remained significantly associated with higher self-reported competence. In contrast, academic degrees, teaching awards, and article publications were not statistically significant.

Finally, the binary logistic regression model, which used the 50th percentile of the S-CONE score as a cut-off point, revealed that supervision of undergraduate theses, participation in scientific conferences and involvement in projects as the most consistent predictors of high self-reported teaching competence (see Table 3).

## 4. Discussion

The sample, composed of 596 nursing educators, was largely female (85.6%), with a median age of 41.9 years and extensive clinical experience, which coincides with the profiles described internationally. Studies from Australia and New Zealand [2], Finland [13] and Italy [19], and a European multicentre sample [20] reports similar patterns of middle-aged professionals with substantial experience. In contrast, studies from China [10] and Vietnam [21] describe younger educators and earlier transitions into teaching roles, illustrating how access to clinical teaching varies considerably by context.

Most of the available studies describe the characteristics of nurse educators or explore bivariate relationships between isolated variables, but they have not explored multivariate associations between teaching competence and the sociodemographic or professional profiles of clinical educators. In this sense, our study provides a more integrative approach, by identifying the factors associated with high levels of self-reported teaching competence.

Regarding the role played, most of the participants in this study worked as clinical mentors (81.4%), while 10.2% had a mixed role (clinical mentor and academic tutor). This predominance of the clinical profile may reflect the composition of the sample, focused on university hospitals linked to clinical teaching. Our sample contrasts with that of McAllister [2], where mixed roles were more frequent. Our study sample is more similar to the one observed in Finland and Italy, where most educators also have a clinical role [13,19]. Asian literature also shows diversity: In Japan, the role of the clinical instructor is stressed in clinical learning [22], while, in Iran, researchers developed a specific instrument for the evaluation of clinical teaching performance, arguing that the systematic evaluation of this function constitutes a key strategy for ensuring the quality of teaching and guiding professional development [23]. Regarding academic level, almost half of the participants had master’s degrees (47.8%), although the number of doctorates was limited. This pattern coincides with the European trend towards progressive postgraduate training [19,20], but contrasts with English-speaking countries, where a doctorate is typically required for an academic career in nursing. In Australia and New Zealand, researchers found that 18% of nurse educators had a doctorate and 48% a master’s degree [2], while in the United States the National League for Nursing and other organisations consider the doctorate to be a basic credential for access to university academic positions. However, these requirements apply above all to academic university training, and in clinical teaching the requirements differ significantly. In this sense, a comparative study showed that in most European countries, clinical educators have a bachelor’s or master’s degree, and the doctorate is not a formal requirement, although there is variability in terms of professional experience and the pedagogical training required [24]. This finding is consistent with our results, in which, despite the high proportion of people with postgraduate degrees, most of the participants (73.8%) had not received formal pedagogical training, which indicates a key area for improvement.

Together, these results suggest that, although the sociodemographic and professional characteristics of clinical nursing educators are somewhat homogeneous internationally, there may be relevant differences in access to teaching, the balance between clinical and academic functions, and teacher training opportunities. These observations should be interpreted with caution, since they could be conditioned by the characteristics of the sampling procedure and the participating institutions. However, they provide useful clues for the design of strategies that strengthen professional development in specific contexts.

### 4.1. Teaching Competence Levels

Most rated themselves as competent in their teaching work, with high scores in all factors of the S-CONE questionnaire. Particularly noteworthy are those related to professional practice, curricular design, and the incorporation of evidence. Among the items with the highest level of self-reported competence are providing constructive feedback, guiding reflective practice, and recognizing the value of research in teaching.

However, areas for improvement were identified. The lowest scores corresponded to capabilities such as activating the interest of the student, presenting complex information, applying innovative strategies, and reading scientific literature on a regular basis. These aspects, linked to advanced teaching, presented a lower proportion of professionals who rated themselves as competent. This pattern may reflect not only individual skill gaps, but also structural and organisational constraints such as limited protected time for pedagogical innovation, restricted access to educational resources, and weak integration between clinical, teaching, and research activities. This pattern has already been described in previous studies, which highlight the tendency of nurse educators to perceive themselves as more competent in communicative and supervisory tasks than in innovative aspects of educational practice [25,26,27]. Capabilities related to innovation or the integration of evidence often require institutional support and specific training [28,29], and their development may be limited by the lack of coordination between teaching and research or by insufficient initial pedagogical training [30,31]. This may explain the apparent discrepancy observed in our study between high interest in research participation and lower self-reported competence in the “Research and evidence” domain, suggesting that contextual barriers rather than motivational deficits are at play.

Together, these findings suggest that although the overall level of teaching competence was high, specific areas should be reinforced through training strategies oriented towards critical thinking, pedagogical innovation, and the incorporation of scientific evidence in clinical teaching.

### 4.2. Relationship Between Teaching Competence and Sociodemographic, Professional, Training, and Research Variables

The bivariate analysis showed that self-reported teaching competence was significantly related to professional and academic variables. Nurses with a mixed role (clinical mentor and academic tutor) or an academic tutor role scored themselves higher than those with exclusively clinical functions, which coincides with international studies that point to simultaneous exposure to care and academic contexts as a factor that enhances teaching competence [13,20]. Pedagogical training, both general and specific in clinical teaching, was also associated with higher levels of competence, in line with reviews confirming that structured training strengthens teacher self-efficacy and the ability to evaluate learning [4,32]. Likewise, academic and research activities (conference presentations, research projects, and the supervision of undergraduate theses) were consistently associated with higher scores on the S-CONE, reinforcing the idea that academic productivity acts as a driver of teacher development, as indicated by recent multicentre studies [33].

In contrast, no differences were found based on sex, age, professional experience, or teaching experience, which contrasts with studies carried out in other contexts. For example, in a Nigerian cohort, Ogunmuyiwa et al. identified female gender as a predictor of greater teaching competence [34], while in an Indian sample, Pareek et al. pointed to age as an associated factor [33]. The absence of associations in our study could be due to the low variability of these variables in the sample analysed, which limits their explanatory capacity.

Multiple linear regression models confirmed that academic productivity was the block with the highest capacity to explain the variance of teaching competence (adjusted R^2^ = 0.170), followed by the educational role (adjusted R^2^ = 0.094). These results are consistent with previous studies that have documented the impact of academic and scientific involvement on the development of teaching skills [35,36]. Consistently, the binary logistic regression analysis identified scientific communications and thesis supervision as the strongest predictors of high self-reported competence, replicating findings that place the integration of research and teaching as a key factor of educational excellence [33].

Together, these results suggest that clinical nurses’ perception of their teaching competence is mainly related to pedagogical training and involvement in academic and research activities, rather than to sociodemographic attributes or accumulated professional experience. These findings reinforce that pedagogical competence and research engagement are not independent domains but mutually reinforcing components of clinical teaching capability. This supports the need for institutional strategies that actively promote faculty development, pedagogical training, and integration of teaching, research, and clinical practice as core components of quality clinical education. It is possible that the professionals most involved in research or teaching tasks perceived a greater mastery of their abilities, which could reflect a self-efficacy effect and/or a greater real development of skills. The convergence between our findings and the international literature reinforces the recommendation to incorporate structured teacher training programmers and to promote academic productivity as key strategies to strengthen professional development and the quality of clinical teaching in nursing.

### 4.3. Limitations

This study has some limitations that must be taken into account when interpreting the results. First, the cross-sectional design prevents establishing causal relationships between the variables analysed and self-reported teaching competence. Although statistically significant associations have been identified, it is not possible to determine the direction of these relationships or to rule out the influence of factors not included in the analysis. To overcome this limitation, future research could use longitudinal or quasi-experimental designs to explore the evolution of teaching capabilities over time or assess the impact of specific training interventions.

Second, data collection using a self-report questionnaire may have introduced social desirability or perception of biases. This may partially explain the generally high levels of self-reported competence observed, particularly in domains related to leadership, professional commitment, and research engagement. It is possible that some participants overestimated or underestimated their level of teaching competence, especially in more abstract or self-referential factors such as pedagogical innovation or research. The inclusion of other sources of information, such as direct observations, student evaluations, or qualitative triangulation, could provide a more complete and objective perspective in future studies.

Although the study is multicentric and includes a large sample from different health centres, the results are limited to a single regional health system. Therefore, they may not be directly transferable to contexts with different organisational models, regulatory frameworks, or clinical education structures. This circumstance limits the generalisability of the findings to other care or educational contexts with different organisational structures and professional development models. Comparative studies between regions or countries could help validate these results in different settings.

Another limitation involves the presence of variables with low frequency, such as the authorship of books or the publication of scientific articles, which may have had limited explanatory power in statistical models. Although a block approach was chosen to facilitate their gradual inclusion, their low representation may have affected the stability and significance of the coefficients. The use of targeted sampling techniques or Bayesian models could be useful in future research to treat these types of variables.

Finally, the study did not consider organisational, cultural, or contextual variables of the work environment, such as institutional support, care burden, or access to training resources, which could play a role in the development and maintenance of teaching competence. The incorporation of these factors in future work, through mixed methods or structural equation modelling, could make it possible to obtain a more holistic understanding of the phenomenon studied.

Taken together, these limitations invite a prudent interpretation of the results. At the same time, they open up new lines of research that could contribute to designing more precise strategies for the development and recognition of teaching competence in clinical settings.

## 5. Conclusions

This study shows that nurses involved in the teaching of clinical placement subjects perceive themselves as having an overall high level of teaching competence, with particularly strong scores in professional practice and curriculum design, and relatively lower scores in the incorporation of scientific evidence. At the same time, relevant areas for improvement were identified, especially in pedagogical innovation and the systematic use of scientific evidence, which require targeted educational and organisational support. The perception of teaching competence in this sample was mainly associated with teaching role, pedagogical training, and involvement in academic activities, whereas personal characteristics such as clinical experience and sex appeared to play a more limited role.

Although these findings are derived from a specific regional and institutional context, they highlight patterns that are likely to be relevant for other clinical education systems with similar organisational structures. Taken together, the results suggest that the development of teaching competence cannot rely solely on individual motivation or accumulated clinical experience but requires explicit institutional commitment to faculty development. This includes the implementation of structured pedagogical training programmes, the recognition of teaching and academic engagement in professional career pathways, and organisational conditions that facilitate the integration of clinical practice, teaching, and research activities. These findings support the relevance of institutional policies that actively promote and sustain teaching competence in clinical settings as a core component of educational quality and patient care.

Future research should further explore organisational and contextual determinants of teaching competence and examine its evolution over time through longitudinal and mixed methods approaches to inform more effective strategies for strengthening clinical education across diverse health and academic systems.

## Figures and Tables

**Table 1:** Sociodemographic, professional, training, and research characteristics of clinical nurse educators (n = 596).

Variable		n	%
Age group	<29 years	80	13.4
	30–39 years	166	27.9
	40–49 years	190	31.9
	≥50 years	160	26.8
Sex	Female	510	85.6
	Male	86	14.4
Teaching role	Clinical mentor	485	81.4
	Academic tutor	32	5.4
	Nurse teacher	18	3.0
	Mixed (clinical mentor and academic tutor)	61	10.2
Workplace	Badalona Serveis Assistencials	6	1.0
	Consorci Sanitari Integral	38	6.4
	Fundació Hospital Esperit Sant	85	14.3
	Fundació Sanitària Mollet	5	0.8
	Universitat de Barcelona	17	2.9
	Hospital Sagrat Cor	38	6.4
	Hospital Universitari Bellvitge	184	30.9
	Hospital Universitari Germans Trias i Pujol	72	12.1
	Hospital Universitari Mútua de Terrassa	119	20.0
	Hospital Universitari Viladecans	32	5.4
Clinical unit	Not identified	128	21.5
	Hospitalisation	207	34.7
	Emergency	148	24.8
	ICU*/Surgical Block	63	10.6
	Other	50	8.4
Education level	Diploma	206	34.6
	Bachelor’s/Licentiate	99	16.6
	Master’s	285	47.8
	Doctorate	6	1.0
Teaching award	Yes	64	10.7
	No	532	89.3
Teacher training	Yes	156	26.2
	No	440	73.8
Training in clinical education	Yes	133	22.3
	No	463	77.7
Years of nursing experience	1–10 years	153	25.7
	11–20 years	197	33.1
	21–30 years	157	26.3
	>30 years	89	14.9
Articles published (last 5 years)	None	523	87.8
	1–3 articles	61	10.2
	>3 articles	12	2.0
Books published (last 5 years)	None	572	96.0
	1–3 books	22	3.7
	>3 books	2	0.3
Conference presentations (last 5 years)	None	350	58.7
	1–3	182	30.5
	>3	64	10.7
Pedagogy courses (last 5 years)	None	465	78.0
	1–3	116	19.5
	>3	15	2.5

ICU*: Intensive Care Unit.

**Table 2:** Bivariate analysis of S-CONE overall scores by sociodemographic, professional, training, and research variables (n = 596).

Variables	Median (IQR) *^a^*	*p*
**Sex *^b^***		
Male	253.5 (48.5)	0.482
Female	258 (54.5)	
**Age ^c^**		
<29 years	260 (46.5)	0.88
30–39 years	261 (60.2)	
40–49 years	257 (52.7)	
>50 years	257 (54.7)	
**Academic degree ^c^**		
Diploma	248 (53.2)	<0.001
Bachelor’s/Licentiate	257 (57)	
Master’s	264 (54.5)	
Doctorate	327 (27.2)	
**Educator role ^c^**		
Clinical mentor	252 (53)	<0.001
Academic tutor	291 (27)	
Nurse teacher	246 (55)	
Mixed (clinical mentor and academic tutor)	280 (47.5)	
**Nursing experience *^c^***		
1–10 years	260 (50.5)	0.629
11–20 years	259 (58.5)	
21–30 years	258 (54)	
>30 years	254 (50)	
**Teaching experience ^c^**		
1–10 years	261 (55.7)	0.896
11–20 years	257 (54)	
21–30 years	254 (55)	
>30 years	257 (93.5)	
**Teacher training ^b^**		
Yes	273 (53.5)	<0.001
No	253 (52)	
**Training in clinical education ^b^**		
Yes	276 (56)	<0.001
No	254 (52)	
**Pedagogy courses ^c^**		
None	254 (53)	<0.001
Between 1 and 3	270 (51.7)	
More than 3	303 (75)	
**Teaching award ^c^**		
Si	284.5 (47.5)	<0.001
No	256 (52)	
**Projects carried out ^c^**		
None	247 (49.2)	<0.001
Between 1 and 3	274 (48)	
More than 3	284 (45)	
**Undergraduate theses supervised ^b^**		
None	256 (53.5)	<0.001
Between 1 and 3	275.5 (55)	
More than 3	301 (48)	
**Articles published *^b^***		
None	255 (53)	<0.001
Between 1 and 3	282 (52)	
More than 3	284 (55.2)	
**Books published ^c^**		
None	257 (54)	0.164
Between 1 and 3	277 (55.5)	
More than 3	277.5	
**Conference presentations ^c^**		
None	248 (52)	<0.001
Between 1 and 3	274 (48)	
More than 3	277 (51.7)	
**Department or unit ^b^**		
Not specified	254 (54)	0.004
Hospitalisation	265 (52)	
Emergency	253 (64.7)	
ICU^n^/Surgery	247 (40)	
Other	268 (49.5)	
**Workplace *^c^***		
Badalona Serveis Assistencials	253 (51.5)	<0.001
Consorci Sanitari Integral	270 (66.5)	
Fundació Sanitària Mollet	256 (121)	
Hospital Universitari Mútua de Terrassa	250 (46)	
Hospital Universitari Viladecans	237 (46.3)	
Hospital Universitari Bellvitge	266.5 (52.8)	
Hospital Universitari Germans Trias i Pujol	247 (57.3)	
Fundació Hospital Esperit Sant	257 (58)	
Hospital Sagrat Cor	249 (45)	
Universitat de Barcelona	286 (31)	

^a^ Results are presented as median and interquartile range due to the non-parametric nature of the test; ^b^ Hypothesis test performed using the Mann–Whitney U test with a significance level of *p* < 0.05; ^c^ Hypothesis test performed using the Kruskal–Wallis test with a significance level of *p* < 0.05, adjusted using the Bonferroni correction for multiple comparisons.

**Table 3:** Logistic regression models by factor and total score of the S-CONE questionnaire.

Predictors	OR	95% CI	*p*-Value
**F1. Nursing practice**			
Constant	1.089		0.691
Nursing experience	0.997	[0.973–1.021]	0.799
Teaching experience	1.027	[1.000–1.059]	0.061
Teaching role: academic tutor vs. clinical mentor	2.186	[0.918–5.205]	0.077
Teaching role: nurse teacher vs. clinical mentor	0.739	[0.273–1.997]	0.551
Teaching role: mixed (tutor/mentor) vs. clinical mentor	1.750	[0.881–3.476]	0.110
Teacher training	1.028	[0.575–1.840]	0.925
Training in clinical education (yes, no)	0.774	[0.417–1.438]	0.418
Pedagogy courses	0.982	[0.764–1.261]	0.885
Conference presentations	1.087	[1.014–1.165]	0.018 *
Published articles	0.952	[0.809–1.121]	0.558
Undergraduate theses supervised	0.992	[0.894–1.101]	0.887
Projects carried out	1.077	[0.955–1.213]	0.228
Observations	596		
R^2^/R^2^ adjusted	0.039/0.052		
**F2. Curriculum design and implementation**			
Constant	1.089		0.626
Nursing experience	0.997	[0.973–1.021]	0.799
Teaching experience	1.027	[0.999–1.057]	0.061
Teaching role: academic tutor vs. clinical mentor	2.186	[0.918–5.205]	0.077
Teaching role: nurse teacher vs. clinical mentor	0.739	[0.273–1.997]	0.551
Teaching role: mixed (tutor/mentor) vs. clinical mentor	1.750	[0.881–3.476]	0.110
Teacher training (yes, no)	1.028	[0.575–1.840]	0.925
Training in clinical education (yes, no)	0.775	[0.417–1.438]	0.418
Pedagogy courses	0.982	[0.764–1.261]	0.885
Conference presentations	1.087	[1.014–1.165]	0.018 *
Published articles	0.952	[0.809–1.121]	0.558
Undergraduate theses supervised	0.992	[0.894–1.101]	0.887
Projects carried out	1.076	[0.955–1.213]	0.228
Observations	596		
R^2^/R^2^ adjusted	0.052/0.039		
**F3. Communication, collaboration, and partnership**			
Constant	0.325		0.216
Nursing experience	0.999	[0.975–1.023]	0.957
Teaching experience	1.031	[1.003–1.062]	0.031
Teaching role: academic tutor vs. clinical mentor	2.517	[1.040–6.727]	0.045 *
Teaching role: nurse teacher vs. clinical mentor	0.763	[0.275–2.060]	0.609
Teaching role: mixed (tutor/mentor) vs. clinical mentor	1.835	[0.908–3.781]	0.082
Teacher training (yes, no)	1.079	[0.608–2.014]	0.778
Training in clinical education (yes, no)	0.798	[0.408–1.391]	0.390
Pedagogy courses	1.006	[0.804–1.307]	0.948
Conference presentations	1.056	[0.987–1.129]	0.105
Published articles	0.993	[0.858–1.178]	0.933
Undergraduate theses supervised	0.995	[0.899–1.096]	0.931
Projects carried out	1.058	[0.942–1.181]	0.312
Observations	596		
R^2^/R^2^ adjusted	0.036/0.048		
**F4. Management, leadership, and advocacy**			
Constant	0.079		0.688
Nursing experience	1.004	[0.980–1.028]	0.748
Teaching experience	1.024	[0.994–1.055]	0.118
Teaching role: academic tutor vs. clinical mentor	2.061	[0.855–4.866]	0.104
Teaching role: nurse teacher vs. clinical mentor	0.801	[0.313–2.274]	0.662
Teaching role: mixed (tutor/mentor) vs. clinical mentor	1.600	[0.798–3.382]	0.170
Teacher training	1.115	[0.615–1.984]	0.724
Training in clinical education	0.832	[0.442–1.531]	0.500
Pedagogy courses	0.981	[0.758–1.247]	0.868
Conference presentations	1.082	[1.004–1.156]	0.036 *
Published articles	0.969	[0.842–1.148]	0.684
Undergraduate theses supervised	0.995	[0.899–1.097]	0.930
Projects carried out	1.070	[0.948–1.197]	0.258
Observations	596		
R^2^/R^2^ adjusted	0.037/0.049		
**F5. Research and evidence**			
Constant	0.930		0.691
Nursing experience	0.997	[0.973–1.021]	0.799
Teaching experience	1.027	[1.000–1.059]	0.061
Teaching role: academic tutor vs. clinical mentor	2.186	[0.918–5.205]	0.077
Teaching role: nurse teacher vs. clinical mentor	0.739	[0.273–1.997]	0.551
Teaching role: mixed (tutor/mentor) vs. clinical mentor	1.750	[0.881–3.476]	0.110
Teacher training	1.028	[0.575–1.840]	0.925
Training in clinical education	0.774	[0.417–1.438]	0.418
Pedagogy courses	0.982	[0.764–1.261]	0.885
Conference presentations	1.087	[1.014–1.165]	0.018 *
Articles published	0.952	[0.809–1.121]	0.558
Undergraduate theses supervised	0.992	[0.894–1.101]	0.887
Projects carried out	1.077	[0.955–1.213]	0.228
Observations	596		
R^2^/R^2^ adjusted	0.039/0.052		

S-CONE: the Spanish version of the Capabilities of Nurse Educators questionnaire; * *p* < 0.05 considered statistically significant; R^2^: Cox and Snell R-square. Adjusted R^2^: Nagelkerke R-square.

## Data Availability

The data presented in this study are not publicly available at this time because they are part of an ongoing doctoral thesis and are subject to an institutional embargo until the thesis has been formally defended and deposited. After the embargo period, data may be available from the corresponding authors upon reasonable request and subject to prior approval by the participating institutions and ethics committees.

## Supplementary Materials

The following supporting information can be downloaded at: https://www.mdpi.com/article/10.3390/nursrep16020041/s1, Table S1. Level of competence per item of the S-CONE questionnaire*.

## Public Involvement Statement

No public involvement in any aspect of this research.

## Guidelines and Standards Statement

This manuscript was drafted against the STROBE (Strengthening the Reporting of Observational Studies in Epidemiology) statement for cross-sectional studies.

## Abbreviations

The following abbreviations are used in this manuscript:

AUC	Area under the curve
ICU	Intensive care unit
S-CONE	Spanish version of the Capabilities of Nurse Educators
UB	University of Barcelona
WHO	World Health Organisation

## References

[1] World Health Organization (2016). Nurse Educator Core Competencies.

[2] McAllister M., Flynn T. (2016). The Capabilities of Nurse Educators (CONE) questionnaire: Development and evaluation. Nurse Educ. Today.

[3] Nussbaum M. (2000). Women and Human Development: The Capabilities Approach.

[4] Lee S.L., Rees C.E., O’Brien B.C., Palermo C. (2022). Identities and roles through clinician-educator transitions: A systematic narrative review. Nurse Educ. Today.

[5] Melnyk B.M., Gallagher-Ford L., Zellefrow C., Tucker S., Thomas B., Sinnott L.T., Tan A. (2018). The first U.S. study on nurses’ evidence-based practice competencies indicates major deficits that threaten healthcare quality, safety, and patient outcomes. Worldviews Evid.-Based Nurs..

[6] Hagrass H.M., Ibrahim S.A.E.A., Anany R.I.E.S., El-Gazar H.E. (2023). Effect of an educational program about mentorship competencies on nurse mentors’ performance: A quasi-experimental study. BMC Nurs..

[7] Mathisen C., Heyn L.G., Jacobsen T.I., Bjørk I.T., Hansen E.H. (2022). The use of practice education facilitators to strengthen the clinical learning environment for nursing students: A realist review. Int. J. Nurs. Stud..

[8] Keinänen A.L., Lähdesmäki R., Juntunen J., Tuomikoski A.M., Kääriäinen M., Mikkonen K. (2023). Effectiveness of mentoring education on health care professionals’ mentoring competence: A systematic review. Nurse Educ. Today.

[9] Rodríguez-Soberado M.P., Martin-Gil B., Fernández-Castro M. (2023). Self-perceived competences in evidence-based practice of clinical-teaching nurses versus clinical nurses. Enferm. Clín. (Engl. Ed.).

[10] He H., Zhou T., Zeng D., Ma Y. (2021). Development of the competency assessment scale for clinical nursing teachers: Results of a Delphi study and validation. Nurse Educ. Today.

[11] Hsu L.L., Hsieh S.I., Chiu H.W., Chen Y.L. (2014). Clinical teaching competence inventory for nursing preceptors: Instrument development and testing. Contemp. Nurse.

[12] Kaarlela V., Mikkonen K., Pohjamies N., Ruuskanen S., Kääriäinen M., Kuivila H.M., Haapa T. (2022). Competence of clinical nurse educators in university hospitals: A cross-sectional study. Nord. J. Nurs. Res..

[13] Tuomikoski A.M., Ruotsalainen H., Mikkonen K., Miettunen J., Kääriäinen M. (2018). Development and psychometric testing of the nursing student mentors’ competence instrument (MCI): A cross-sectional study. Nurse Educ. Today.

[14] Wu X.V., Chi Y., Selvam U.P., Devi M.K., Wang W., Chan Y.S., Wee F.C., Zhao S., Sehgal V., Ang N.K.E. (2020). A Clinical Teaching Blended Learning Program to Enhance Registered Nurse Preceptors’ Teaching Competencies: Pretest and Posttest Study. J. Med. Internet Res..

[15] Hulley S., Cummings S., Browner W., Grady D., Newman T. (2013). Designing Clinical Research.

[16] Chen Y.L., Hsu L.L., Hsieh S.I. (2012). Clinical nurse preceptor teaching competencies: Relationship to locus of control and self-directed learning. J. Nurs. Res..

[17] Li A.T., Su Y.W. (2014). Exploring the relationship between personality features and teaching self-efficacy in clinical nursing preceptors. J. Nurs. Res..

[18] Shin S., Kang Y., Hwang E.H., Kim J. (2021). Factors associated with teaching efficacy among nurse educators in hospital settings. J. Clin. Nurs..

[19] Comparcini D., Cicolini G., Simonetti V., Mikkonen K., Kääriäinen M., Tomietto M. (2020). Developing mentorship in clinical practice: Psychometric properties of the Mentors’ Competence Instrument. Nurse Educ. Pract..

[20] Mikkonen K., Tomietto M., Cicolini G., Kučič B.M., Filej B., Riklikiene O., Juskauskiene E., Vizcaya-Moreno M.F., Pérez-Cañaveras R.M., De Raeve P. (2020). Development and testing of an evidence-based model of mentoring nursing students in clinical practice. Nurse Educ. Today.

[21] Nguyen V.N.B., Forbes H., Mohebbi M., Duke M. (2017). Development and validation of an instrument to measure nurse educator perceived confidence in clinical teaching. Nurs. Health Sci..

[22] Fukuta D., Ishitsuka T., Suzuki J., Mori C. (2019). Developing a short form of the Japanese version of a scale assessing clinical nursing instructors’ effective clinical teaching behaviors. Int. Med. J..

[23] Farahani M.A., Emamzadeh Ghasemi H.S., Nikpaima N., Fereidooni Z., Rasoli M. (2015). Development and psychometric evaluation of the Nursing Instructors’ Clinical Teaching Performance Inventory (NICTPI). Glob. J. Health Sci..

[24] Dobrowolska B., McGonagle I., Kane R., Jackson C.S., Kegl B., Bergin M., Cabrera E., Cooney-Miner D., Di Cara V., Dimoski Z. (2016). Patterns of clinical mentorship in undergraduate nurse education: A comparative case analysis of eleven EU and non-EU countries. Nurse Educ. Today.

[25] Ren R., Chen G., Yan J., Zhang S., Tan J., Yue J.J. (2024). Development and validation of a core competence instrument for clinical nursing teachers: A mixed-methods study. Nurse Educ. Today.

[26] Tuomikoski A.M., Ruotsalainen H., Mikkonen K., Kääriäinen M. (2020). Nurses’ experiences of their competence at mentoring nursing students during clinical practice: A systematic review of qualitative studies. Nurse Educ. Today.

[27] Ahmed G.W., Abd el-rahman S.M., Abood S.A., Abd el-baset M.T. (2019). Clinical instructor’s behaviors as perceived by themselves, students and nursing faculty staff. Minia Sci. Nurs. J..

[28] Buanz S.F., Alsenayien A.Y., Altharman H.A., Alnaqi R.I., Llaguno M.B.B., Mousa O., Siraj R.A. (2024). Nursing students, faculty, and preceptors perception of effective characteristics of clinical instructor: A cross-sectional study. SAGE Open Nurs..

[29] Zhou F., Yuan T., Li Z., Mu X., Lv Y. (2024). The evidence-based practice teaching competence of clinical preceptors at different stages of the innovation-decision process: A cross-sectional survey in traditional Chinese medicine hospitals. Nurse Educ. Today.

[30] Dejene D., Stekelenburg J., Versluis M., Ayalew F., Molla Y. (2022). Assessment of core teaching competency of health professional educators in Ethiopia: An institution-based cross-sectional study. BMJ Open.

[31] Pohjamies N., Haapa T., Kääriäinen M., Mikkonen K. (2022). Nurse preceptors’ orientation competence and associated factors: A cross-sectional study. J. Adv. Nurs..

[32] Oprescu F., McAllister M., Duncan D., Jones C. (2017). Professional development needs of nurse educators: An Australian case study. Nurse Educ. Pract..

[33] Pareek B., Soni M., Rana R., Sharma S., Goyal P., Sharma S. (2023). Nurse educators’ professional practice attributes and its determinants. J. Educ. Health Promot..

[34] Ogunmuyiwa A.O., Anyama S.C., Howells B.B., Ogunleye C.A., Esan D.T. (2025). Gender and age differential on competence level in clinical practice among nursing students in a tertiary institution in Nigeria. SAGE Open Nurs..

[35] Griffiths M., Creedy D.K., Carter A.G. (2021). Systematic review of tools to measure preceptors’ perceptions of their role in undergraduate health clinical education. Nurse Educ. Today.

[36] Pramila-Savukoski S., Juntunen J., Tuomikoski A.M., Kääriäinen M., Tomietto M., Kaučič B.M., Filej B., Riklikiene O., Vizcaya-Moreno M.F., Perez-Cañaveras R.M. (2020). Mentors’ self-assessed competence in mentoring nursing students in clinical practice: A systematic review of quantitative studies. J. Clin. Nurs..

