# Supplementary material for: Nurse Educators’ Self-Reported Level of Teaching Competence and Its Correlation with Sociodemographic, Professional, Training and Research Variables: A Cross-Sectional Multicentre Study
Source: Nurs Rep. 2026 Jan 27;16(2):41. doi: 10.3390/nursrep16020041 (PMC12942969; doi:10.3390/nursrep16020041)
Supplement: Supplementary file 1 [file nursrep-16-00041-s001.zip › nursrep-4033059-supplementary.pdf]

## S-CONE instrument

The second instrument was the S-CONE, the validated Spanish version of the CONE questionnaire developed by McAllister et al. S-CONE consists of 69 items distributed across five factors (nursing practice; curriculum design and implementation; communication, collaboration, and partnership; management, leadership and promotion; and research and evidence), evaluated on a five-point Likert scale (1 = not representative, 5 = very representative). The instrument showed high internal consistency (Cronbach's $\alpha = 0.96$) and adequate temporal stability (ICC > 0.80 for all factors, 15-day test–retest). Construct validity was examined by exploratory factor analysis, using principal component extraction and varimax rotation, after confirming the adequacy of the sample with a KMO index greater than 0.90 and a significant Bartlett sphericity test ($p < .001$). The results indicated a five-factor solution with eigenvalues of 24.73, 6.93, 3.29, 2.34 and 1.87, respectively, which together explained 56.78% of the total variance. All the items included presented factor loads greater than 0.40 in their corresponding dimension, and no relevant cross-loading was observed, confirming the theoretical and statistical coherence of the questionnaire. Teaching competence scores were obtained as means by dimension and overall. For the classification, the theoretical midpoint of the Likert scale (1–5) was used, equivalent to a value of 3.0. Scores equal to or greater than 3.0 were considered competent, and scores below 3.0 were considered not competent.

**Table S1.** Level of competence per item of the S-CONE questionnaire*.

| Dimension/Item | Competent n (%) | Not competent n (%) | Mean (SD) |
|---|---|---|---|
| *Nursing practice* | | | *32.22 (4.29)* |
| 1. Broad knowledge of nursing practice | 578 (97.0%) | 18 (3.0%) | 4.04 (0.77) |
| 2. Proper use of clinical practices | 576 (96.6%) | 20 (3.4%) | 4.03 (0.74) |
| 53. Recognises importance of research | 595 (99.8%) | 1 (0.2%) | 4.57 (0.55) |
| 54. Disseminates research findings | 582 (97.7%) | 14 (2.3%) | 4.06 (0.76) |
| 55. Believes research enhances learning | 557 (93.5%) | 39 (6.5%) | 3.82 (0.82) |
| 59. Research is essential for practice | 589 (98.8%) | 7 (1.2%) | 4.08 (0.70) |
| 61. Supports inquiry culture | 584 (98.0%) | 12 (2.0%) | 4.07 (0.73) |
| 62. Frequently reads scientific articles | 538 (90.3%) | 58 (9.7%) | 3.54 (0.86) |
| *Curriculum design and implementation* | | | *89.37 (18.42)* |
| 3. Listens empathetically | 429 (72.0%) | 167 (28.0%) | 3.15 (1.17) |
| 5. Uses various communication strategies | 539 (90.4%) | 57 (9.6%) | 3.81 (0.90) |
| 6. Plans learning experiences | 424 (71.1%) | 172 (28.9%) | 3.08 (1.10) |
| 7. Manages situational problems | 406 (68.1%) | 190 (31.9%) | 3.01 (1.11) |
| 8. Activates student interest | 355 (59.6%) | 241 (40.4%) | 2.82 (1.13) |
| 9. Offers innovative solutions | 385 (64.6%) | 211 (35.4%) | 2.94 (1.14) |
| 11. Fosters learning opportunities | 545 (91.4%) | 51 (8.6%) | 3.78 (0.91) |
| 17. Guides reflective practice | 559 (93.8%) | 37 (6.2%) | 3.85 (0.85) |
| 18. Asks reflective questions | 434 (72.8%) | 162 (27.2%) | 3.16 (1.13) |
| 20. Follows effective teaching practices | 546 (91.6%) | 50 (8.4%) | 3.89 (0.93) |
| 21. Plans formative evaluations | 509 (85.4%) | 87 (14.6%) | 3.55 (1.00) |
| 22. Explains problem-solving strategies | 562 (94.3%) | 34 (5.7%) | 3.97 (0.84) |
| 23. Provides constructive feedback | 585 (98.2%) | 11 (1.8%) | 4.25 (0.73) |
| 25. Plans assessment activities | 405 (68.0%) | 191 (32.0%) | 3.04 (1.18) |

| | | | |
|---|---|---|---|
| 29. Creates a positive environment | 517 (86.7%) | 79 (13.3%) | 3.50 (0.90) |
| 30. Presents complex information clearly | 362 (60.7%) | 234 (39.3%) | 2.80 (1.09) |
| 31. Develops adaptable strategies | 391 (65.6%) | 205 (34.4%) | 2.95 (1.13) |
| 33. Identifies areas for improvement | 351 (58.9%) | 245 (41.1%) | 2.72 (1.15) |
| 35. Provides supportive environment | 582 (97.7%) | 14 (2.3%) | 4.32 (0.74) |
| 36. Uses counselling strategies | 553 (92.8%) | 43 (7.2%) | 3.93 (0.86) |
| 37. Answers student questions | 550 (92.3%) | 46 (7.7%) | 3.75 (0.87) |
| 38. Prepares students for clinical practice | 339 (56.9%) | 257 (43.1%) | 2.73 (1.20) |
| 39. Inspires excellence with vision | 474 (79.5%) | 122 (20.5%) | 3.36 (1.04) |
| 40. Understands learning in context | 454 (76.2%) | 142 (23.8%) | 3.25 (1.09) |
| 41. Confident in teaching ability | 555 (93.1%) | 41 (6.9%) | 3.97 (0.87) |
| 43. Demonstrates dynamism | 544 (91.3%) | 52 (8.7%) | 3.79 (0.91) |
| *Communication, collaboration, and partnership* | | | 33.95 (4.53) |
| 44. I have a positive attitude | 584 (98.0%) | 12 (2.0%) | 4.05 (0.72) |
| 47. I am empathetic | 586 (98.3%) | 10 (1.7%) | 4.15 (0.74) |
| 48. I am approachable | 571 (95.8%) | 25 (4.2%) | 4.13 (0.83) |
| 49. I listen attentively | 586 (98.3%) | 10 (1.7%) | 4.30 (0.75) |
| 50. I accept feedback from superiors | 577 (96.8%) | 19 (3.2%) | 4.20 (0.80) |
| 58. I advocate for evidence-based practice | 582 (97.7%) | 14 (2.3%) | 4.21 (0.76) |
| 68. I enjoy teaching | 589 (98.8%) | 7 (1.2%) | 4.41 (0.70) |
| 81. I reflect regularly to facilitate academic practice | 593 (99.5%) | 3 (0.5%) | 4.50 (0.66) |
| *Management, leadership, and advocacy* | | | *72.77 (8.28)* |
| 10. I can participate in research projects | 595 (99.8%) | 1 (0.2%) | 4.57 (0.59) |
| 12. I can disseminate research findings | 595 (99.8%) | 1 (0.2%) | 4.55 (0.60) |
| 13. I can identify research methodologies | 592 (99.3%) | 4 (0.7%) | 4.56 (0.62) |
| 15. I can manage projects | 577 (96.8%) | 19 (3.2%) | 4.25 (0.84) |
| 16. I can evaluate research outcomes | 572 (96.0%) | 24 (4.0%) | 4.26 (0.86) |

| | | | |
|---|---|---|---|
| 19. I promote nursing research | 594 (99.7%) | 2 (0.3%) | 4.73 (0.55) |
| 24. I can distinguish research articles | 587 (98.5%) | 9 (1.5%) | 4.40 (0.72) |
| 26. I use different research methodologies | 580 (97.3%) | 16 (2.7%) | 4.41 (0.80) |
| 27. I formulate research questions | 584 (98.0%) | 12 (2.0%) | 4.27 (0.75) |
| 28. I apply theoretical frameworks | 394 (66.1%) | 202 (33.9%) | 3.00 (1.18) |
| 32. I conduct research studies | 595 (99.8%) | 1 (0.2%) | 4.75 (0.50) |
| 34. I contribute to the nursing profession | 568 (95.3%) | 28 (4.7%) | 4.16 (0.90) |
| 65. I respect students | 525 (88.1%) | 71 (11.9%) | 3.84 (1.06) |
| 85. I participate in interdisciplinary academic discussions | 588 (98.7%) | 8 (1.3%) | 4.49 (0.68) |
| 86. I belong to professional nursing bodies | 587 (98.5%) | 9 (1.5%) | 4.33 (0.74) |
| 87. I frequently support students | 563 (94.5%) | 33 (5.5%) | 4.12 (0.92) |
| 92. I maintain interprofessional networks | 596 (100.0%) | 0 (0.0%) | 4.70 (0.50) |
| 93. I foster respect between educators and students | 559 (93.8%) | 37 (6.2%) | 3.95 (0.88) |
| *Research and evidence* | | | *28.20 (7.55)* |
| 69. I seek opportunities to improve my teaching | 353 (59.2%) | 243 (40.8%) | 2.88 (1.30) |
| 70. I care about students' progress | 329 (55.2%) | 267 (44.8%) | 2.77 (1.27) |
| 71. I promote the importance of interprofessional relationships | 205 (34.4%) | 391 (65.6%) | 2.20 (1.44) |
| 72. I reflect on my teaching practice | 561 (94.1%) | 35 (5.9%) | 4.09 (0.94) |
| 73. I consider myself responsible | 461 (77.3%) | 135 (22.7%) | 3.47 (1.28) |
| 82. I enjoy researching | 359 (60.2%) | 237 (39.8%) | 2.89 (1.34) |
| 89. I build university relationships | 350 (58.7%) | 246 (41.3%) | 2.86 (1.34) |
| 90. I maintain collaborative networks | 251 (42.1%) | 345 (57.9%) | 2.41 (1.35) |
| 91. I am interested in research participation | 593 (99.5%) | 3 (0.5%) | 4.63 (0.64) |
| ***S-CONE overall*** | | | **256.53 (37.32)** |

* A score equal to or greater than 3 was considered to reflect an acceptable or high level of competence, based on the theoretical midpoint of the S-CONE Likert scale (1–5). Item numbers correspond to the original CONE questionnaire item identifiers; S-CONE includes 69 items after item reduction.
